# Impact of Non-Confinement Accommodation on Farrowing Performance: A Systematic Review and Meta-Analysis of Farrowing Crates Versus Pens

**DOI:** 10.3390/ani9110957

**Published:** 2019-11-12

**Authors:** Dannielle Glencorse, Kate Plush, Susan Hazel, Darryl D’Souza, Michelle Hebart

**Affiliations:** 1 SunPork Group, PO Box 92, Wasleys 5400, Australia; 2 School of Animal and Veterinary Science, Faculty of Sciences, The University of Adelaide, Roseworthy 5371, Australia

**Keywords:** farrowing pen design, piglet mortality, stillborn, farrowing accommodation, non-confinement, systematic review, meta-analysis, sow

## Abstract

**Simple Summary:**

The aim of this project was to review previously published research with a focus on the effects of farrowing accommodation on piglet performance. The specific design features were analysed to determine whether animals in loose housed farrowing pens or crates from loading to weaning contribute to differences in litter performance obtained from different farrowing house accommodation types. This was the first systematic review and meta-analysis conducted towards the farrowing performance of crates and pens. The relative risk of piglet mortality was 14% higher in farrowing pens than farrowing crates, which indicated that non-confinement of sows compromises post-natal piglet survival. Overall, the type of farrowing accommodation did not affect the number of stillborn piglets. However, the rate of stillborn piglets was lower in farrowing pens that were not enriched when compared with farrowing crates, also with no enrichment. There was no effect of housing type on the number of piglets born alive or the number of piglets weaned, although the sample size for the later was much smaller. Producers should anticipate an increase in mortality when piglets are reared by sows that are unconfined in the pen designs that are currently available, which supports the wider belief that crates are successful for reducing pre-weaning piglet mortality.

**Abstract:**

There are conflicting reports regarding the effect of farrowing house accommodation on piglet performance. The aim of this investigation was to use a systematic review and meta-analyses to summarise the results of publications that focused on direct comparisons between full confinement conventional crates and various designs of loose-housed farrowing pens from loading until weaning. Literature searches in Scopus, BIOSIS Previews, Cab Abstracts, and Web of Science identified 6695 articles. Twenty-two publications were retained for the systematic review and individual meta-analyses after screening for inclusion criteria. The random effects meta-analyses were performed on crate versus pen for number of piglets born alive, number of stillborn piglets, pre-weaning mortality, and number of piglets weaned. Additionally, the modifiers of confinement length (no confinement from loading until weaning or partial confinement for shorter periods of time in the early stages post parturition), enrichment (no enrichment or enrichment provided), and pen size (small, medium, or large) were examined. There was a 14% increase in the relative risk of piglet mortality in farrowing pens when they were compared with crates (*p* = 0.0015). The number of stillborns per litter was not different between the pen and crate. However, when providing enrichment in the pens, there was an increase in stillborns within farrowing crates versus pens (*p* = 0.009). There was no overall effect on piglets that were born alive or number weaned. As there is no difference between piglets born alive and mortality is significantly higher in farrowing pens, a reduction in the number of piglets weaned was expected but not observed, which was possibly due to the lack of weaning details provided in the publications. This was the first systematic review and meta-analysis conducted on the performance of farrowing accommodation and identified that farrowing pens do compromise post-natal piglet survival. Future efforts should focus on improving sow comfort in the farrowing crate to maximize both piglet and sow welfare.

## 1. Introduction

Intensive animal production often comes under review because of a range of practices that are considered to be adverse for the animals involved [1]. The welfare issues encountered when pigs are intensively reared are related to overcrowding, restrictive space allocations, barren environments, and the isolation of individual animals [2]. The farrowing crate has been criticized, as it imposes several of these welfare issues on the periparturient and lactating sow [3,4,5]. The farrowing crate was introduced to intensive pig breeder farms with several aims: to reduce piglet mortality from sow crushing, provide a clean and hygienic environment for neonatal piglets to grow, and protect stock-people from sow aggression [1,5]. Farrowing crates were initially devised with the aim of providing a safe working environment and maintaining pre-wean mortality as low as 10% [6]. Despite these benefits, there is evidence that housing sows in farrowing crates leads to compromised sow welfare, as confinement results in an increased stress response at certain times during farrowing and lactation [3,7,8]. Repetitive, bar-associated behaviours or stereotypies often develop prior to and during parturition as sows attempt to form nest areas in a restrictive environment [9]. As a result, housing options that reduce the level of sow confinement during parturition and lactation have received attention in recent literature.

A significant concern for producers is that non-confinement of sows in farrowing pens leads to an increase in exactly what farrowing crates were designed to minimise: piglet mortality. Piglet mortality that was associated with crushing increased by 6–9% when the sows farrowed in open, confinement free pens versus farrowing crates [6]. Whilst some investigations report exactly this [6,10], the results from others suggest that the type of farrowing system results in little influence on piglet mortality [11]. The reason as to why similar results have been achieved in pens and crates is unclear, but is most likely explained by a range of influences that would include design features, management procedures, sow factors, environmental factors, and experimental design flaws, such as insufficient statistical power [1,5,12,13]. This inconsistency has led to limited large-scale commercial pen adoption, which is anticipated to continue until success factors related to farrowing pens are better understood. 

The meta-analysis is a statistical tool that allows for the combination of results across multiple scientific studies and allows for the determination of important factors that affect key variables across experiments [14]. A meta-analysis was applied to 45 experiments reported in 42 publications to determine the relative importance of pen design features in grower pig housing. From this, these authors determined the importance of factors, such as space allowance, enrichment, and group size on behaviour, growth, and efficiency which contribute to sustainable production [4]. A similar methodology has been applied to gestation housing in sows [15], which was able to evaluate physiology, behaviour, and production outcomes from multiple publications where group and stall housing were compared. To date, there is no published evidence of such an analysis on lactation sow housing. Thus, the aim of this investigation was to conduct a systematic review to identify the key variables that may impact on piglet output, and then perform a meta-analysis on these included publications to determine the factors that result in the comparable performance of farrowing pens. We hypothesized that overall, piglet mortality would be higher in pens than crates, but that there would be specific features of pens that result in similar performance.

## 2. Materials and Methods 

Guidelines for conducting a systematic review were obtained from Wylie et al. [16]. This publication complies with the publication guidelines that were provided by Preferred Reporting Items for Systematic Reviews and Meta-Analysis (PRISMA) [14]. 

### 2.1. Search Strategy

The literature searches were conducted on the 21st of February 2018 in four databases; Scopus, BIOSIS Previews, Cab Abstracts, and Web of Science. A search protocol was designed to obtain any articles that provided data on sow farrowing performance and the design of farrowing accommodation. The search terms used were identified as being relevant by the authors and designed broadly to ensure that all publications conducted in farrowing pens were included. The specific terms varied based on the database in question, but all of the methods included the terms ‘farrow’ AND ‘sow’ AND ‘design’ OR ‘housing’ OR ‘system’. The actual search frames that were used for each database are provided below: 

Search method for Scopus

TITLE-ABS-KEY (farrow* AND sow AND (design OR housing OR system))—856 results

Search method for BIOSIS previews 

TS = (farrow* AND sow AND (design OR housing or system))—2546 results

Search method for Cab Abstracts

TS = (farrow* AND sow AND (design OR housing or system))—2330 results

Search method for Web of Science

TS = (farrow* AND sow AND (design OR housing or system))—961 results

### 2.2. Screening & Eligibility

Searching the four databases identified a total of 6695 articles. All of the articles collected from the online searches were downloaded to Endnote (X7.7.1). Hard copy conference proceedings from the Australasian Pig Science Association (APSA) and final reports from research supported by the Co-operative Research Centre for High Integrity Australian Pork in Australia were imported into the database and screened accordingly. The reference lists of included studies were scanned for potential new inclusions while using the study eligibility form. All of the publications were sorted by the first author, title, and abstract of the articles screened to remove duplicates, and a species scan was conducted to remove articles that did not focus on pigs. An inclusion/exclusion checklist was developed to identify papers conducted in a research area relevant to the hypothesis (Table 1). The abstract and full paper were investigated to further examine studies with relevant titles. If there was uncertainty on whether an individual publication complied with the inclusion and exclusion criteria, a decision was made by discussion with all authors. If a publication was not accessible as a full text, the corresponding author was contacted with a follow-up email that was sent two weeks after the initial email if no response was received. The paper was excluded from the study if the authors did not respond within four weeks of the first contact. 

### 2.3. Inclusion and Exclusion Criteria

A publication was selected if the experimental design included a comparison of a non-confinement in a farrowing environment at any point from parturition to weaning with traditional farrowing crates. In addition, the methodology was required to include details on the design of the farrowing area and to measure piglet performance (mortality rate, number of stillborn piglets, and number of weaned piglets). Any study that involved outdoor, free range, or group housing during parturition and lactation was excluded. Publications that were written in a language other than English were excluded if a translatable version was not accessible. Similarly, articles that were published prior to 1990 were excluded, if there were no accessible copies. 

### 2.4. Data Collection

If the inclusion criteria were satisfied, data from each publication collected in the format that is outlined in Table 2. All data was present in the included publications and as a result, no authors were contacted for obtaining additional data that was no published in the original article. 

### 2.5. Quality Assessment 

A quality assessment form was adapted (Table 3) to enable the analysis of the quality of each publication through a weighted comparison of separate studies [16]. The rating system provided two individual scores; the first score measured the quality of generic experimental design and the second score quantified the specific details of the farrowing environment. These two scores were combined, and each article was assigned a rating out of 36, with a higher score indicating that the experiment was robust and relevant to the topic of this review. One reviewer conducted the quality scoring on all of the publications that were included in the systematic review and meta-analysis.

### 2.6. Meta-Analysis

The extracted data were analysed in individual meta-analysis models while using four data subgroups; number of piglets born alive (n = 28), number of stillborn piglets (n = 27), total piglet mortality from parturition to weaning (n = 30), and number of piglets weaned (n = 15). The sample size varied for each analysis, based on the data that were published in each article. The data were represented as the total number present in each litter, rather than as a percentage of total born or born alive piglets, with sow being an experimental unit. Any articles that reported these values as a percentage of total born or born alive were transformed while using the reported litter sizes to a value that represented a total number per litter. Each data-point included in the meta-analysis demonstrated a comparison between a farrowing crate and one type of pen. If there was more than one pen-type included in an article, they were included as separate data-points. 

### 2.7. Statistical Analyses

Random effects meta-analyses were performed on the database, while using the metaphor package R statistical software Version 3.2.5 (R Core Team, Vienna, Austria) to examine whether pooled effect sizes for crate versus pen housing altered number of piglets born alive, number piglets stillborn, pre-weaning mortality, and number of piglets weaned [17]. A pooled estimate of the mean relative risk of these traits and the corresponding 95% confidence intervals were calculated by random effects logistic regression model (binomial-normal model) to allow for heterogeneity in the analysis. Each measure of piglet viability was examined in separate meta-analyses to compare the effect of farrowing environment (pen or crate) by considering individual inter-publication variation. The measure of heterogeneity (I2) indicated the variation between studies. The measures of piglet mortality and number of stillborns were analysed while using relative risk. Risk, as opposed to odds, is calculated as the number of piglets in the group who died divided by the total number of piglets in the group. A relative risk greater than one indicated increased likelihood of the stated outcome being achieved in the treatment group, less than one indicated there was a decreased likelihood in the treatment group, and a ratio of one indicated no difference, that is the outcome is just as likely to occur in the treatment group as it is in the control group. The born alive and weaned number of piglets were continuous variables and, hence, were analysed while using standardized mean difference (SMD). Estimates of the traits and their associated confidence intervals were calculated by transforming the mean log-risk and its confidence interval back into the probability scale. The Q-test was used to assess statistical heterogeneity between studies and the I2 was calculated to describe the amount of inconsistency of findings across studies. Post hoc exploratory meta-regression analyses were performed, which included enrichment (provided or not provided), confinement (no confinement from loading until weaning or partial confinement for shorter periods of time in the early stages post parturition), and pen area (small, medium and large) to evaluate potential moderators to explain heterogeneity. The presence or absence of straw as a source of enrichment for sows within farrowing accommodation was recorded. Each pen environment was classified as small, medium, or large with ranges of 2.8–4.9 m^2^ (similar range to conventional farrowing crate), 5–7.5 m^2^ (equivalent size of farrowing pens as defined in the Animal Welfare Code of Practice), and greater than 7.5 m^2^ (greater space allocation than farrowing pen defined in the Animal Welfare Code of Practice), respectively. Funnel plots to test the asymmetry and publication bias of individual studies were conducted [18]. A *p*-value of less than 0.05 was considered to be significant.

## 3. Results

### 3.1. Database Search

**Figure 1:**
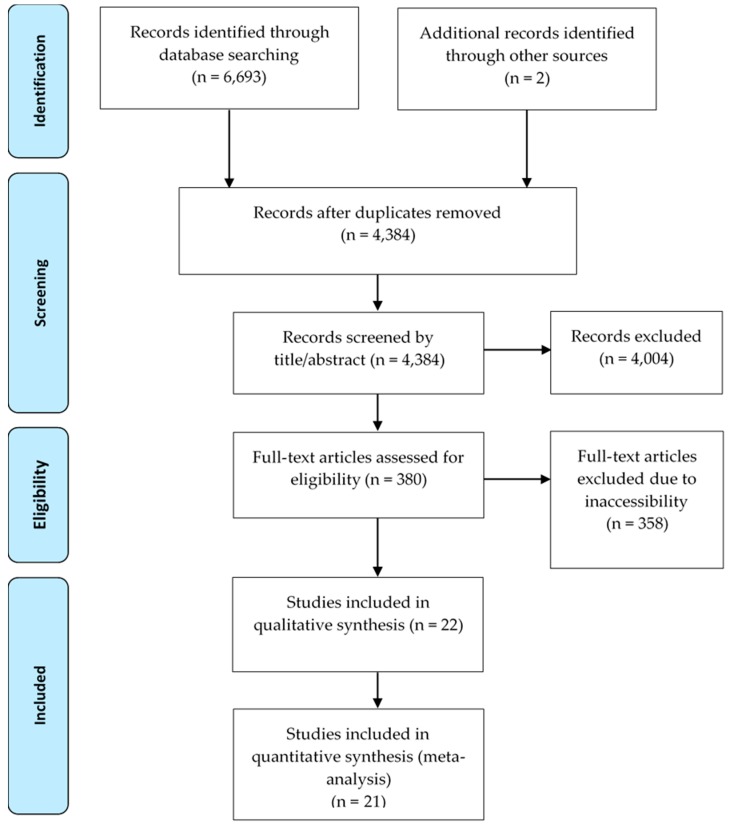
A flow diagram documenting the process of identification and screening of eligible publications for inclusion in the systematic review and meta-analysis of non-confinement farrowing accommodation design. The flow chart was adapted from [16].

The database search identified 6693 articles from four databases and two articles from external sources. After the duplicates were removed, there were 4483 articles remaining. Of these publications, 380 were deemed to be relevant when the inclusion and exclusion criteria were applied to the title. Twenty-two of these articles were examined in detail to enable the extraction of information for systematic review. The systematic review identified 32 individual comparisons between a farrowing crate and a farrowing pen from the twenty-two publications. Figure 1 presents a flow diagram of the publications that are involved in the screening and eligibility and the data used in each meta-analysis are shown in Appendix A. Data were extracted from 4385 litters, with data provided from 2182 and 2203 sows farrowing in crates and pens respectively. The articles were conducted in a range of countries, including Australia, Canada, China, Czech Republic, Denmark, Finland, Germany, New Zealand, United Kingdom, and USA, and were published from 1990 to 2016. Only 14% of articles were abstracts, conference proceedings or final reports while 86% of publications were peer-reviewed, full journal articles. The average sample size for the included studies was 68 (range = 6–394). Sample size was often restricted in these publications with 40% involving less than 20 sows per treatment while 22% of studies were conducted with more than 60 animals per treatment. The specific type of pen (e.g., Freedom) was not assessed, as the sample size for each type was too small for analysis. Studies using loose-housed farrowing pen systems from loading to weaning were compared to studies within farrowing pens that provided an option for confinement for a short period during parturition and early lactation. 

### 3.2. Quality Assessment Scoring 

A quality assessment score was provided for each publication. The scores associated with each publication for the twenty-two quality assessment criteria are presented below (Table 4). The overall quality of all the publications was high with half of the publications obtaining a score greater than 70% (range = 25–31) and 95% of publications obtaining a score greater than 50% (range = 19–31). Only one publication was assigned a score of less than 50%. The primary aim of 72.7% of publications was to compare the traditional farrowing crate with a non-confinement farrowing accommodation from loading until weaning. The remaining articles focused on behaviour analysis as the primary focus while still providing farrowing performance data. Over 81% of publications involved a comparison between two housing types, while 18.2% involved an experimental design with more than three treatments. The dimensions of each housing type, with distinctions for the separate sow and creep areas, were provided for 86.4% of publications, while the remaining publications provided minimal detail on either total area or total size. The design of the farrowing environment was described in varied detail. The lighting conditions were only described in 4.5% of publications, while creep area heating was defined in all but one of the articles. The flooring material in the creep and sow areas were described in all the publications whereas ventilation and environmental design of sheds was clearly described in only 27.3% of the articles. Only 36.4% of publications described the components in detail of the farrowing space that would enable the protection of piglets or restraint of the sow. However, there were still over 90% of publications that mentioned the presence of these protective designs. Enrichment was provided for sows in 68.2% of the studies that were included in the review. Over 59% of the experiments were designed for both farrowing crates and pens to be housed in the same rooms. Clear protocols and definitions were provided for fostering in 50% of publications. However, within the top 12 studies, 75% provided clear definitions of the methods for fostering. Similarly, while only 27.3% of publications provided detailed piglet mortality definitions and values, 68.2% of studies separated piglet mortality into smaller sub-groups that were based on the cause of death. Overall, the quality of data collection for farrowing and weaning performance was moderate with only 50% and 27.3% of publications, respectively, providing comprehensive figures. 

### 3.3. Qualitative Systematic Review

The systematic review identified 28 articles that reported the number of piglets born alive in each litter. The average number of piglets born alive was 12.54 in both farrowing crates (range = 8.4–17.1) and farrowing pens (range = 8.8–17.1). The results in 50% of articles indicated a decrease in piglets that were born alive in farrowing pens, 32% indicated an increase in born alive and the remaining articles found no change between farrowing housing types in piglets born alive.

Twenty-seven articles recorded the number of stillborn piglets for farrowing pens and crates. Stillborn piglets were recorded as a total number of stillborns in all litters for each farrowing accommodation type. Crates resulted in 77.81 stillborns (range = 5–416) across all litters, while 81.37 (range = 8–440) stillborns were found in farrowing pens. Over 59% of articles reported a decrease in the number of stillborns that were found within farrowing pens, while 37% of published work indicated the contradictory result, an increase in stillborns in pens.

Pre-weaning mortality was recorded as a total number of piglet deaths in each farrowing accommodation across all litters. An increase in pre-weaning mortality in farrowing pens was observed in 56% of publications, while 40% of published data indicated that the farrowing crates had more piglet mortality. The average pre-weaning mortality for farrowing crates was 101.66 piglets/trial (range = 7–506), which was lower than 123 piglets/trial (range = 9–590) in farrowing pens.

The number of piglets weaned per litter was published in 15 articles. The number weaned was 9.73 piglets/litter in farrowing crates (range = 7.1–12) and 9.81 piglets/litter in farrowing pens (range = 7.54–12.3). One-third of articles indicated a decrease in the number of piglets weaned/litter in farrowing pens, 46% measured an increase, while 20% recorded no difference between housing types. 

### 3.4. Meta Analysis

**Figure 2:**
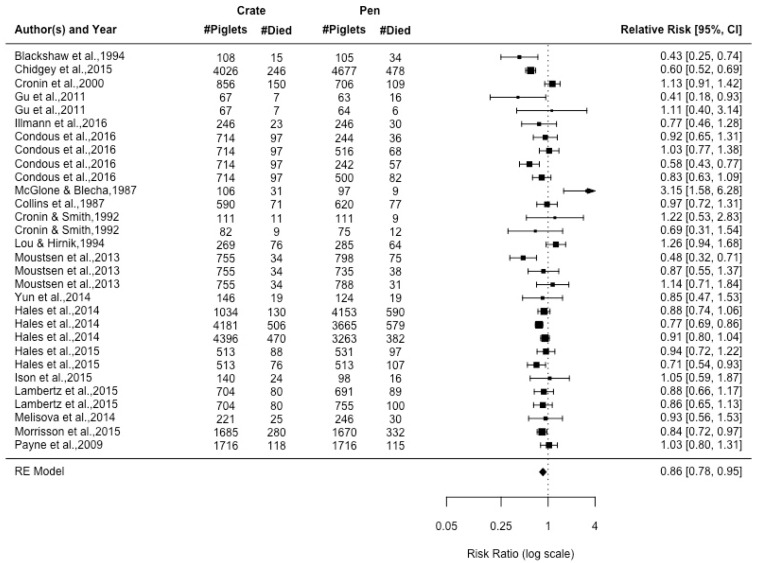
Forest plot of piglet mortality displaying relative risk for all articles are presented with 95% confidence interval. Each line represents an article or individual comparison between a farrowing crate and a farrowing pen alternative. A relative risk greater than 1 indicates increased likelihood of the piglet mortality being achieved in a farrowing crate relative to farrowing pen.

**Figure 3:**
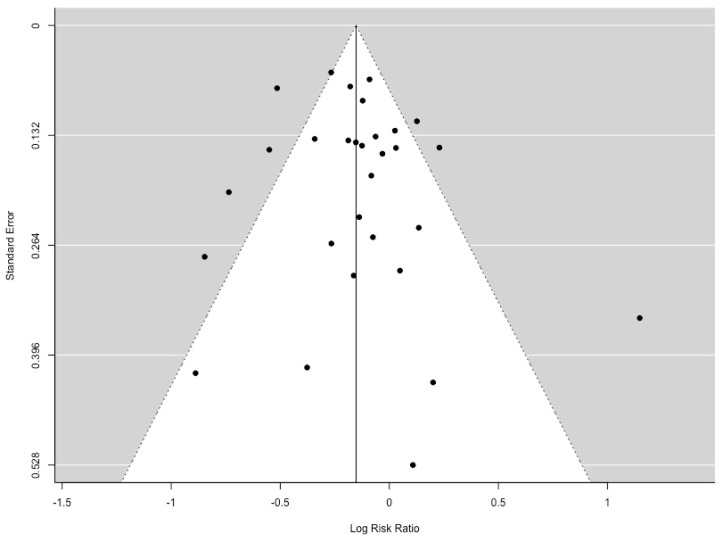
Funnel plot for all publications included in meta-analysis of total piglet mortality (n = 30). Grey areas indicate publications of significance, the dotted line represents the relative risk of 1 shown by a line of no-effect and white areas show non-significance. If no publication bias is present, the data-points will be organized symmetrically.

The data used for the meta-analyses is reported in the Appendix A (Table A1 and Table A2). Total piglet mortality had a moderate amount of heterogeneity (I2 = 69.81%, *p* = 0.002). The relative risk of piglet mortality in a farrowing crate was 14% lower than a farrowing pen (Figure 2). Several external factors were examined as moderators. There was no effect of confinement type (whether sows were unconfined for the entire lactation period or had partial confinement; *p* = 0.853), sow enrichment with straw (*p* = 0.801), or relative pen size (*p* = 0.206) on pre-weaning mortality in penned sows. A funnel plot regression test that is shown in Figure 3 indicates that there was no publication bias (z = 0.538, *p* = 0.591). 

**Figure 4:**
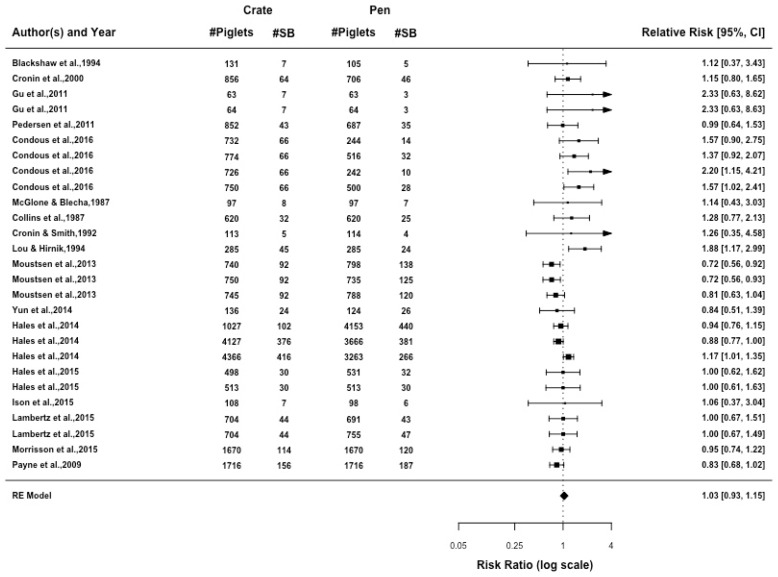
Forest plot of number of stillborn piglets displaying relative risk for all articles is presented with 95% confidence interval. Each line represents an article or individual comparison between a farrowing crate and a farrowing pen alternative. A relative risk greater than 1 indicates increased likelihood of stillborn piglets being achieved in farrowing crates relative to farrowing pens.

**Figure 5:**
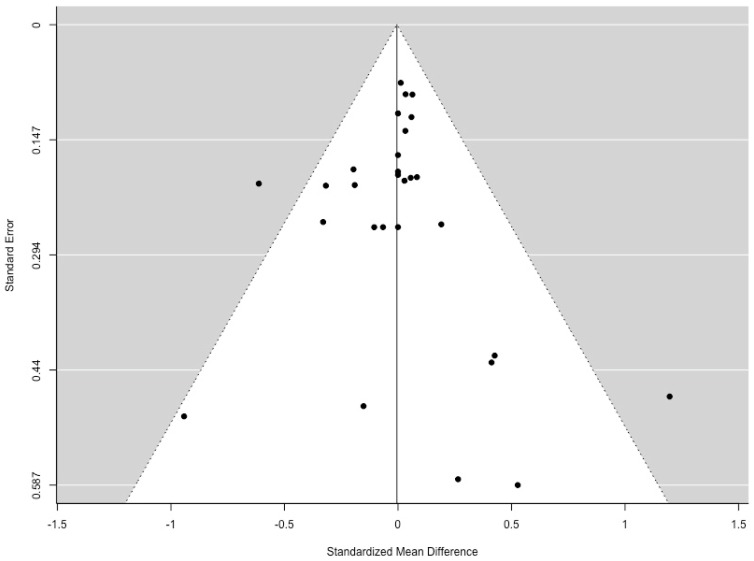
Funnel plot for all publications included in meta-analysis of number of stillborn piglets (n = 27). Grey areas indicate publications of significance, the dotted line represents the relative risk of 1 shown by a line of no-effect and white areas show non-significance. If no publication bias is present, the data-points will be organized symmetrically.

The number of stillborn piglets recorded in each litter had a moderate amount of heterogeneity (I2 = 57.93%, *p* = 0.001). The relative risk of stillborn piglets was comparable in farrowing pens and farrowing crates (Figure 4). There was no effect when comparing the confinement type (*p* = 0.706), or between crates and pens when the pen size was considered to be standard (*p* = 0.089). However, when including enrichment as a moderator there was a significant difference between crates and pens. There was no difference in the relative risk of stillborn piglets in crates and pens when enrichment was provided, but when there was no enrichment of the farrowing pen, stillborn piglets in crates were 22% higher than in pens (*p* = 0.009). When enrichment was provided, the relative risk of stillborn piglets was decreased by 10% in crates relative to pens (*p* = 0.099). A funnel plot regression test, as shown in Figure 5, indicates that there is no publication bias (z = −0.223, *p* = 0.823).

**Figure 6:**
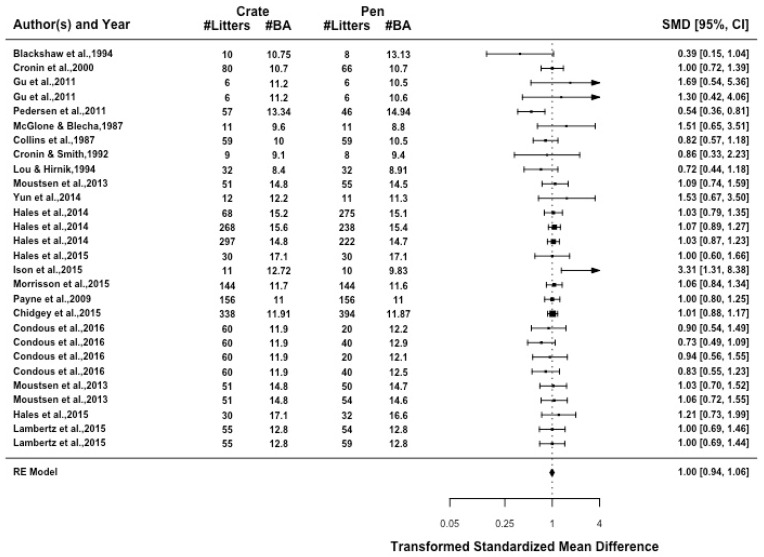
Standardized mean difference (SMD) for all articles are presented with 95% confidence interval. Each line represents an article or individual comparison between a farrowing crate and a farrowing pen alternative. A SMD greater than 1 indicates increased likelihood of born alive piglets being achieved in farrowing pens when compared to farrowing crates.

The number of piglets born alive in each litter had a high amount of heterogeneity (I2 = 0%, *p* = 0.303). The farrowing crates and pens had no difference in the number of piglets born alive (Figure 6) and, within farrowing pens there was no effect of confinement type (*p* = 0.786), sow enrichment (*p* = 0.597) or pen size (*p* = 0.659).

**Figure 7:**
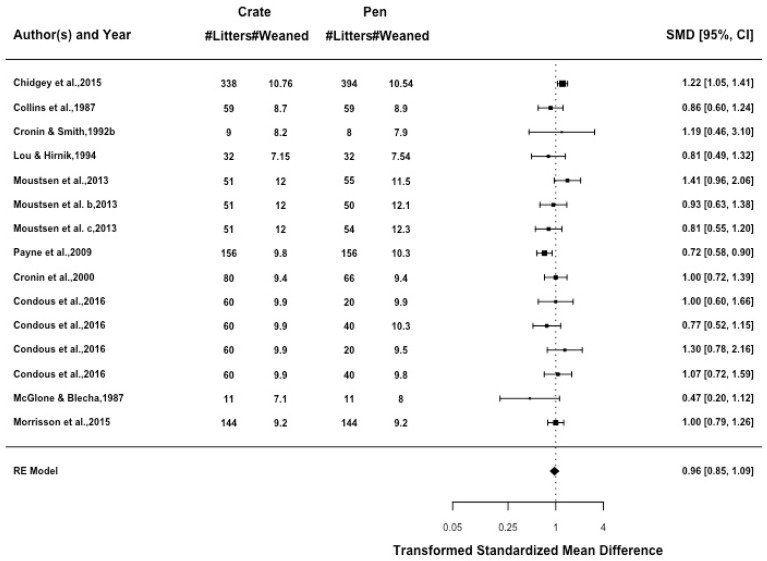
Standardised mean difference (SMD) for all articles are presented with 95% confidence interval. Each line represents an article or individual comparison between a farrowing crate and a farrowing pen alternative. A SMD greater than 1 indicates increased likelihood of wean number being achieved in farrowing pens when compared to farrowing crates.

The number of piglets weaned from each litter had a low amount of heterogeneity (I2 = 43.96%, *p* = 0.021). There was no difference in the number of pigs weaned between crates and pens (Figure 7), and there was no effect when comparing the moderators of confinement type (*p* = 0.567), sow enrichment (*p* = 0.765), or pen size (*p* = 0.333) within pens.

## 4. Discussion

Traditionally, farrowing crates have been the preferred housing type to enhance piglet survivability. The restrictive nature of a farrowing crate prevents sow movement, leading to a reduction in the number of piglets that are crushed by the sow; the primary cause of piglet death [19,20]. The main benefit of a farrowing pen is an increase in the freedom of movement, which ensures that sows can conduct a normal range of behaviour, particularly during farrowing [21]. As the sow undergoes hormonal changes that lead to restlessness and erratic posture changes, the increased range of movement within a pen is expected to be associated with an increase in piglet death due to sow overlay [22,23,24]. The current finding determined that total piglet mortality was 14% more likely in a pen than in a crate supports this notion. Farrowing crates were designed to reduce movement of the sow that could cause overlaying or squashing of piglets [25]. By removing the restrictive structures sows can perform more posture changes and conduct these changes at a greater speed, which heightens the risk of overlays [10,26]. However, there are other factors outside of this that may lead in increases in piglet deaths. Farrowing pens are often larger in size than crates, and so specifically designed areas (creeps), which meet thermal needs of newborn piglets become harder to locate [3]. A crate with a smaller area has fewer spaces that can cause piglet deaths that are associated with exposure to cold temperatures. Most farrowing crates have a separate heated creep area to accommodate for the different temperature requirements for piglets and sows. Ultimately, the farrowing crate was designed to ensure piglet comfort and survival [1,3]. Death from exposure is more likely if a piglet fails to locate the creep. Novel projects that increase the likelihood of piglets remaining in the creep area within a pen may act to limit chilling and the associated deaths that are caused by overlays for non-viable piglets [27]. Additionally, whilst pen size was shown to exert little influence on piglet deaths in our analyses, the numbers of animals used in most investigations were likely too few to examine such impacts on the exact causes of piglet mortality. The last way in which piglet mortality may be increased under pen conditions is the willingness of the stockperson to interact with the sow and her litter when housed in farrowing pens [9]. Farrowing crates provide a safer environment for stock-people to work in, especially during the period immediately following farrowing when hormonal changes in the sow often result in high levels of aggression [7]. Under pen conditions, the reduced confinement and high levels of sow aggression can make stockperson interventions that aimed at improving piglet survival more difficult. One study cited significant farm differences in mortality comparisons between crates and pens [28]. Whilst there would have been animal, environmental, and nutritional differences between farms, personal communication with the authors indicated that the farm with pen performance comparable to crates employed stock-people with exceptional neonatal piglet care skills. Obviously, no detail on the level of stockperson skill was included in any of the publications that were included in this review and meta-analysis, and so we could not objectively examine this variable.

Confinement during the peri-parturient period has been linked to an increased incidence of stillbirth rates [29]. The phenomenon is now commonly referred to as the confinement-stillbirth hypothesis [30]. Investigations into the physiological underpinnings have identified that confined sows exhibit an increased level of cortisol prior to farrowing [31], a decrease in post-expulsion oxytocin pulse [21], as well an extended farrowing duration and inter-piglet birth intervals [32]. However, since its inception, some have refuted the link between sow confinement and the incidence of intra-partum piglet mortality [33]. Additionally, higher salivary cortisol has been observed in loose-housed farrowing accommodation as compared to confined sows [34], with no effect on stillborn rates, farrowing duration, or birth interval [35]. The results from the current meta-analysis would suggest that there is no overall improvement in the incidence of stillbirths in pens when compared with crates. However, when no enrichment was included for farrowing pens, the relative risk of stillborn piglets was 22% lower in farrowing pens versus farrowing crates without enrichment. This cements the idea that simply allowing the sow a greater freedom of movement in combination with a nesting substrate leading up to and during farrowing reduces the risk of intra-partum piglet death. This identifies that the provision of enrichment was beneficial for penned sows [36,37]. The presence of straw in farrowing pens presents a substrate that could be a challenge for piglet movement towards the teats. Small low-viability piglets would have difficulty moving through the straw-based nest. Mortality that is associated with exposure to cold temperatures would increase if the piglets are incapable of moving through the dense bedding [38], while alternate studies suggest that straw provisions can improve temperature regulation for piglets [3]. However, chilling does present an issue with the classification of dead piglets, as difficulty in differentiating between stillborn and exposure-based deaths could lead to false positive classifications. This is due to physical similarities in stillborn, low-viability, and exposure piglets, as well as the positioning of the piglets near the rear of the sow. The refinement of current methods for classification of piglet status when booking-in litter information would be improved through assessment of more vigorous autopsy procedures.

The observed pre-weaning mortality was significantly higher in farrowing pens, and so this would then logically impact on the number of pigs weaned. However, lactation housing bore no impact on how many piglets were weaned. Most piglet deaths occur within the first 36 h post-farrowing [19]. A common husbandry technique that was adopted within farrowing houses is cross-fostering, which involves the movement of piglets from one sow to another [39]. Given that this process generally occurs at 24h after farrowing, any piglets that die prior to this fostering process can be replaced. With this reasoning, pre-weaning mortality can be higher in pens (when it occurs prior to fostering) with the number of pigs weaned remaining constant. The systematic review found that 50% of published literature failed to describe the fostering protocol used and only one article indicated that fostering occurred within treatment. While some publications did describe the protocol implemented, there was a lack of consistency in the reporting of several performance outcomes, which introduced variation in the data presented, making a meta-analysis unfeasible. However, individual piglet viability is variable, with differences in piglet vitality between pen and crate litters [40]. Additionally, fostered piglets that are moved from one litter to another may experience greater stress in the crucial peri-natal period. Future studies should identify whether there are differences in growth potential between fostered and non-fostered piglets in different birth and rearing environments.

Meta-analyses are an innovative tool that can be used to assess overall trends in publications and lead to the instigation of production changes. They have been applied to allow the refinement of housing design and carcass features in pig production [4,41]. There are several areas that could be enhanced by applying this technique to allow for the refinement of animal management, including the assessment of environmental conditions, reproductive management, or tracking disease transmission through herds. However, the limitation in data availability that was identified in the current study must be addressed to maximise the significance of any outcomes. The quality of previous literature is a major limitation for both literature and systematic reviews. The published literature included in the current study often lacked enough detail in the presentation of farrowing accommodation performance data, while also providing limited definitions for calculations used to obtain this data. Future publications should ensure that environmental and farrowing performance data should be more detailed and transparent when assessing any farrowing trials.

This study identified that current farrowing pen designs that are available in commercial production are flawed in one major area—pre-weaning piglet mortality. While other performance outcomes such as piglets born alive and number of piglets weaned remain consistent across pen and crate housing types, there is an increase in piglet mortality. This study determined that the number of piglets born alive does not differ, which indicates that reproductive development of piglets in utero is not affected by housing. Additionally, the management of un-confined sows is sufficient to ensure that stillborn rates do not increase in loose-housing. This suggests that the main concern for future research in farrowing pens should be focused on reducing piglet mortality. Adjusting environmental conditions and the refinement of farrowing pen design can allow for housing that is conducive for piglet survival. A systematic review should be conducted on the intervention strategies that have been used to reduce piglet mortality to determine the viable mechanisms that can be implemented in commercial production.

## 5. Conclusions

This was the first systematic review and meta-analyses conducted into the influence of farrowing pen housing on the piglet traits that are important for farrowing house performance. The relative risk of pre-weaning mortality was 14% higher in farrowing pens when compared with crates. The number of piglets born dead was comparable between the crates and pens with enrichment, but the relative risk of stillbirth was increased by 22% in crates versus pen without enrichment. The number of pigs weaned was unaffected by lactation housing design, but this result could reflect an flawed experimental design and data inclusion in publications.

## Figures and Tables

**Table 1:** Inclusion and exclusion criteria used to screen and identify publications relevant to the topic.

Inclusion Criteria	Exclusion Criteria
Did the publication have farrowing pens included in this study?	Did the publication include group housing accommodation during farrowing?
Did the publication compare non-confinement or partial confinement farrowing accommodation with a traditional farrowing crate?	Did the publication include outdoor housing accommodation during farrowing?
Did the publication include the required outcomes of born alive litter size, piglet mortality rate, number of stillborn piglets and number of piglets weaned?	
Did the publication include any descriptive or visual information on farrowing crate design?	

**Table 2:** List of data that was collected from each publication for the systematic review and meta-analysis.

Data Extracted from Each Publication	
1	Paper title
2	Authors
3	Journal
4	Publication year
5	Country
6	Source of publication—Scopus, BIOSIS Previews, Web of Science, CAB Abstracts
7	Publication type—Journal article, conference proceedings, final report
8	Primary aim
9	Secondary aim
10	Number of farrowing accommodations compared
11	Parity structure of herd
12	Sample size for each experimental group
13	Inclusion of experimental controls
14	Randomisation
15	Standardisation – were experiments conducted in same room/shed or separate site
16	Statistical tests
17	Significance level
18	Length of time housed pre-farrow
19	Total time housed within farrowing accommodation
20	Fostering procedures
21	Mortality recording procedures
22	Supervision procedure
23	Assistance procedure
24	Area of farrowing space available to sow
25	Area of farrowing space allocated to creep
26	Total area of farrowing space
27	Creep area features—flooring, heating, shape, materials, lid, enrichment
28	General pen features—flooring, lighting, ventilation, materials, enrichment
29	Piglet protection designs
30	Farrowing details—Total born, born alive, stillborn, mummified piglets
31	Mortality records
32	Weaning details - Number of piglets, litter weights

**Table 3:** Quality assessment scoring system that was used to assess the quality of experimental.

Quality Assessment Questions		Score 0	Score 1	Score 2
A1	How was the study reported?	Unpublished, non-peer reviewed	Abstract, conference proceeding	Full paper
A2	Was the study population representative of a general population (e.g., range of parity)?	No	Yes, the population was described or consisted of one parity group	Yes, population described and included range of parities
A3	Was the sample size sufficient?	<20 sows per treatment	20–60 sows per treatment	>60 sows per treatment
A4	Was the control group appropriate?	No, not present	Partially, not well selected	Yes
A5	Were appropriate statistical tests conducted?	No	Simple inferential statistics or incorrect methods used	Yes, multivariable analysis
A6	Were conclusions made based on statistical significance (*p* < 0.05 or less)?	No	Yes	
A7	Was the experimental design randomised?	No	Yes	
B1	Did this study aim to compare the effect of accommodation on farrowing performance?	No	Yes, secondary aim	Yes, primary aim
B2	How many suitable accommodation types were compared?		Two	Three
B3	Were the dimensions/area of the accommodation provided?	No	Yes, total area provided	Yes, creep and sow areas defined separate
B4	Were the lighting conditions described?	No	Yes	
B5	Were the heating conditions described?	No	Yes	
B6	Were the flooring/mat conditions described?	No	Yes	
B7	Were the ventilation conditions described?	No	Yes	
B8	Were the piglet protection/sow restraint measures described?	No	Yes	Described in detail or pictures provided
B9	Were the enrichment conditions described?	No	Yes	
B10	Were the pre-farrow times provided?	No	Yes	
B11	Were the total housing lengths provided?	No	Yes	
B12	Were the fostering protocols described?	No	Yes	
B13	Were the mortality definitions described?	No	Yes, limited definitions and values provided	Yes, detailed definitions and values provided
B14	Were the comprehensive farrowing details (total born, born alive, born dead, mummified) provided?	One detail provided	Two details provided	≥Three details provided
B15	Were the weaning details (number, weight, average weight) provided?	None	One detail provided	≥Two details provided
B16	Were the farrowing designs located in different physical locations/rooms/sheds?	Different sheds	Same sheds, different room	Same shed, same room

**Table 4:** Percentage of publications that were assigned scores for the quality assessment of 22 criteria, separated for all studies and for the top 12 studies according to the highest rating.

	Top 12 STUDIES			All Studies		
Criterion	Score 0 (%)	Score 1 (%)	Score 2 (%)	Score 0 (%)	Score 1 (%)	Score 2 (%)
A1	4.5	9.1	86.4	8.3	8.3	83.3
A2	18.2	31.8	50	8.3	41.7	50
A3	40.9	36.4	22.7	16.7	41.7	41.7
A4	0	9.1	90.9	0	8.3	91.7
A5	0	45.5	54.5	0	41.7	58.3
A6	4.5	95.5	NA	0	100	NA
A7	0	100	NA	0	100	NA
B1	0	27.3	72.7	0	0	100
B2	NA	81.8	18.2	NA	75	25
B3	0	13.6	86.4	0	8.3	100
B4	95.5	4.5	NA	91.7	8.3	NA
B5	4.5	95.5	NA	0	100	NA
B6	0	100	NA	0	100	NA
B7	72.7	27.3	NA	50	50	NA
B8	9.1	54.5	36.4	0	41.7	58.3
B9	31.8	68.2	NA	25	75	NA
B10	9.1	90.9	NA	8.3	91.7	NA
B11	31.8	68.2	NA	25	75	NA
B12	50	50	NA	25	75	NA
B13	4.5	68.2	27.3	0	58.3	41.7
B14	22.7	27.3	50	0	25	75
B15	36.4	36.4	27.3	33.3	33.3	33.3
B16	22.7	18.2	59.1	16.7	25	58.3

Refer to Table 3 for the explanation of each criterion. NA is used if the criterion does not have an option for the associated score.

## Appendix A

**Table A1:** Summary for each publication included in the meta-analysis including publication date, country, journal, pen type and pen design features (type of confinement, total pen area and enrichment provided).

First Author (Ref. #)	Year	Country	Journal	Pen Type	Confinement Type	Area	Enrichment
Blackshaw et al. [10]	1994	Australia	Appl Anim Behav Sci	Pen	Full	3.94 m^2^	No
Chidgey et al. [42]	2015	New Zealand	Livest Sci	Combi-Flex turn around pen	Partial	5.85 m^2^	No
Collins et al. [43]	1987	U.S.A	Appl Anim Behav Sci	Hillside	Full	3.57 m^2^	No
Condous et al. [30]	2016	Australia	J Anim Sci	Swing-sided pen	Partial	6.02 m^2^	No
Condous et al. [30]	2016	Australia	J Anim Sci	Swing-sided pen	Partial	6.02 m^2^	No
Condous et al. [30]	2016	Australia	J Anim Sci	Swing-sided pen	Partial	6.02 m^2^	No
Condous et al. [30]	2016	Australia	J Anim Sci	Swing-sided pen	Partial	6.02 m^2^	No
Cronin & Smith [44]	1992a	Australia	Appl Anim Behav Sci	Pen	Full	7.20 m^2^	Yes
Cronin & Smith [45]	1992b	Australia	Appl Anim Behav Sci	Pen	Full	7.20 m^2^	Yes
Cronin et al. [11]	2000	Australia	Aust J Exp Agric	Werribee farrowing pen	Full	8.16 m^2^	Yes
Gu et al. [46]	2011	China	Prev Vet Med	Freedom pen	Full	5.75 m^2^	No
Gu et al. [46]	2011	China	Prev Vet Med	Freedom pen	Full	5.75 m^2^	No
Hales et al. [28]	2014	Denmark	Animal	Pen	Full	5.40 m^2^	No
Hales et al. [28]	2014	Denmark	Animal	Pen	Full	5.20 m^2^	Yes
Hales et al. [28]	2014	Denmark	Animal	Pen	Full	6.30 m^2^	Yes
Hales et al. [35]	2015	Denmark	Livest Sci	Pen	Partial	5.25 m^2^	Yes
Hales et al. [35]	2015	Denmark	Livest Sci	Pen	Partial	5.25 m^2^	Yes
Illmann et al. [47]	2016	Czech Republic	J Anim Sci	Pen	Full	5.88 m^2^	Yes
Ison et al. [48]	2015	U.K	Appl Anim Behav Sci	PigSAFE	Full	9.68 m^2^	No
Lambertz et al. [49]	2015	Germany	Animal	Pen	Full	2.80 m^2^	No
Lambertz et al. [49]	2015	Germany	Animal	Pen	Full	2.80 m^2^	No
Lou & Hirnik [50]	1994	Canada	J Anim Sci	Ellipsoid	Full	3.20 m^2^	No
McGlone & Blecha [51]	1987	U.S.A	Appl Anim Behav Sci	Turn around pen	Full	3.90 m^2^	No
Melišová et al. [52]	2014	Czech Republic	J Anim Sci	Pen	Full	5.88 m^2^	Yes
Morrison et al. [53]	2015	Australia	Pork CRC final report	PigSAFE	Full	8.64 m^2^	No
Moustsen et al. [54]	2013	Denmark	Animal	Combi pen	Full	4.70 m^2^	Yes
Moustsen et al. [54]	2013	Denmark	Animal	Combi pen	Partial	4.70 m^2^	Yes
Moustsen et al. [54]	2013	Denmark	Animal	Combi pen	Partial	4.70 m^2^	Yes
Payne et al. [55]	2009	Australia	Manipulating pig production XII	Ring pen	Full	9.36 m^2^	Yes
Yun et al. [56]	2014	Finland	Livest Sci	Pen	Full	7.04 m^2^	Yes

**Table A2:** Piglet farrowing performance (born alive per sow, number of stillborn piglets, total piglet mortality, number of piglets weaned) for each publication included in the meta-analysis.

First Author (Ref. #)	Year	Farrowing Crate				Farrowing Pen			
		Born Alive	Stillborn	No. Weaned	Total Piglet Mortality	Born Alive	Stillborn	No. Weaned	Total Piglet Mortality
Blackshaw et al. [10]	1994	10.75	7		15	13.13	5		34
Chidgey et al. [42]	2015	11.91		10.76	246	11.87		10.54	478
Collins et al. [43]	1987	10	32	8.7	71	10.5	25	8.9	77
Condous et al. [30]	2016	11.9	66	9.9	97	12.2	14	9.9	36
Condous et al. [30]	2016	11.9	66	9.9	97	12.9	32	10.3	68
Condous et al. [30]	2016	11.9	66	9.9	97	12.1	10	9.5	57
Condous et al. [30]	2016	11.9	66	9.9	97	12.5	28	9.8	82
Cronin & Smith [44]	1992a								12
Cronin & Smith [45]	1992b	9.1	5	8.2	11	9.4	4	7.9	9
Cronin et al. [11]	2000	10.7	64	9.4	150	10.7	46	9.4	109
Gu et al. [46]	2011	11.2	7		7	10.5	3		16
Gu et al. [46]	2011	11.2	7		7	10.6	3		6
Hales et al. [28]	2014	15.2	102		130	15.1	440		590
Hales et al. [28]	2014	15.6	376		506	15.4	381		579
Hales et al. [28]	2014	14.8	416		470	14.7	266		382
Hales et al. [35]	2015	17.1	30		88	17.1	32		97
Hales et al. [35]	2015	17.1	30		76	16.6	30		107
Illmann et al. [47]	2016				23				30
Ison et al. [48]	2015	12.72	7		24	9.83	6		16
Lambertz et al. [49]	2015	12.8	44		80	12.8	43		89
Lambertz et al. [49]	2015	12.8	44		80	12.8	47		100
Lou & Hirnik [50]	1994	8.4	45	7.15	76	8.91	24	7.54	64
McGlone & Blecha [51]	1987	9.6	8	7.1	31	8.8	7	8	9
Melišová et al. [52]	2014				25				30
Morrison et al. [53]	2015	11.7	114	9.2	280	11.6	120	9.2	332
Moustsen et al. [54]	2013	14.8	92	12	34	14.5	138	11.5	75
Moustsen et al. [54]	2013	14.8	92	12	34	14.7	125	12.1	38
Moustsen et al. [54]	2013	14.8	92	12	34	14.6	120	12.3	31
Payne et al. [50]	2009	11	156	9.8	118	11	187	10.3	115
Pedersen et al. [40]	2011	13.34	43			14.94	35		
Yun et al. [56]	2014	12.2	24		19	11.3	26		19
